# Fumarate production with *Rhizopus oryzae*: utilising the Crabtree effect to minimise ethanol by-product formation

**DOI:** 10.1186/s13068-020-1664-8

**Published:** 2020-02-01

**Authors:** Reuben M. Swart, Francois le Roux, Andre Naude, Nicolaas W. de Jongh, Willie Nicol

**Affiliations:** Department of Chemical Engineering, University of Pretoria, Lynnwood Road, Hatfield, 0002 Pretoria South Africa

**Keywords:** Fumarate, Fumaric acid, *Rhizopus oryzae*, Crabtree effect, Ethanol, Immobilised fermentation

## Abstract

**Background:**

The four-carbon dicarboxylic acids of the tricarboxylic acid cycle (malate, fumarate and succinate) remain promising bio-based alternatives to various precursor chemicals derived from fossil-based feed stocks. The double carbon bond in fumarate, in addition to the two terminal carboxylic groups, opens up an array of downstream reaction possibilities, where replacement options for petrochemical derived maleic anhydride are worth mentioning. To date the most promising organism for producing fumarate is *Rhizopus oryzae* (ATCC 20344, also referred to as *Rhizopus delemar*) that naturally excretes fumarate under nitrogen-limited conditions. Fumarate excretion in *R. oryzae* is always associated with the co-excretion of ethanol, an unwanted metabolic product from the fermentation. Attempts to eliminate ethanol production classically focus on enhanced oxygen availability within the mycelium matrix. In this study our immobilised *R. oryzae* process was employed to investigate and utilise the Crabtree characteristics of the organism in order to establish the limits of ethanol by-product formation under growth and non-growth conditions.

**Results:**

All fermentations were performed with either nitrogen excess (growth phase) or nitrogen limitation (production phase) where medium replacements were done between the growth and the production phase. Initial experiments employed excess glucose for both growth and production, while the oxygen partial pressure was varied between a dissolved oxygen of 18.4% and 85%. Ethanol was formed during both growth and production phases and the oxygen partial pressure had zero influence on the response. Results clearly indicated that possible anaerobic zones within the mycelium were not responsible for ethanol formation, hinting that ethanol is formed under fully aerobic conditions as a metabolic overflow product. For Crabtree-positive organisms like *Saccharomyces cerevisiae* ethanol overflow is manipulated by controlling the glucose input to the fermentation. The same strategy was employed for *R. oryzae* for both growth and production fermentations. It was shown that all ethanol can be eliminated during growth for a glucose addition rate of $0.07\,\hbox {g}\,\hbox {L}^{-1}\,\hbox {h}^{-1}$. The production phase behaved in a similar manner, where glucose addition of $0.197\,\hbox {g}\,\hbox {L}^{-1}\,\hbox {h}^{-1}$ resulted in fumarate production of $0.150\,\hbox {g}\,\hbox {L}^{-1}\,\hbox {h}^{-1}$ and a yield of $0.802\,\hbox {g}\,\hbox {g}^{-1}$ fumarate on glucose. Further investigation into the effect of glucose addition revealed that ethanol overflow commences at a glucose addition rate of $0.395\,\hbox {g}\,\hbox {g}^{-1}\,\hbox {h}^{-1}$ on biomass, while the maximum glucose uptake rate was established to be between 0.426 and $0.533\,\hbox {g}\,\hbox {g}^{-1}\,\hbox {h}^{-1}$.

**Conclusions:**

The results conclusively prove that *R. oryzae* is a Crabtree-positive organism and that the characteristic can be utilised to completely discard ethanol by-product formation. A state referred to as “homofumarate production” was illustrated, where all carbon input exits the cell as either fumarate or respiratory $\hbox {CO}_{2}$. The highest biomass-based “homofumarate production”: rate of $0.243\,\hbox {g}\,\hbox {g}^{-1}\,\hbox {h}^{-1}$ achieved a yield of $0.802\,\hbox {g}\,\hbox {g}^{-1}$ on glucose, indicating the bounds for developing an ethanol free process. The control strategy employed in this study in conjunction with the uncomplicated scalability of the immobilised process provides new direction for further developing bio-fumarate production.

## Background

Global factors such as the drive to decrease carbon emissions and the interest in green chemistry has increased the demand for biologically produced chemicals. The four-carbon dicarboxylic acids of the TCA cycle (malate, fumarate and succinate) have received widespread attention over the past two decades as potential bio-based platform molecules for the future chemical industry [1]. Most studies have been performed on the biological production of succinate and since 2012 four commercial startups have employed a fermentative strategy to produce succinic acid [2]. Fumarate differs from succinate in degree of reduction, where the internal double bond of fumarate opens up various downstream possibilities. Fumarate hydrogenation to succinate is a straightforward catalytic step. A potential bulk market for the four-carbon dicarboxylic acids lie in the replacement of petrochemically derived maleic anhydride, a $2.1\,\hbox {Mt}\,\hbox {a}^{-1}$ market [3]. Fumarate is ideally suited for this application, where a high-yielding dehydration step can be employed to obtain maleic anhydride [4]. Fumaric acid also differs from the other four-carbon dicarboxylic acids in having a very low water solubility, a useful property for downstream separation with major potential cost benefits. Currently fumaric acid is used in paper resins, unsaturated polyester resins, animal feeds and in the food and beverage industry [5–8]. Fumaric acid additions to livestock feed can reduce methane excretion by up to 32% [9], a major incentive for reducing greenhouse gas emissions from the agricultural sector. Fumaric acid esters have been found to be an effective treatment for psoriasis and multiple sclerosis [10, 11].

Fermentative production of fumarate is traditionally linked to the fungal genus of *Rhizopus*. Although some genetically manipulated prokaryotes have been shown to excrete fumarate in small quantities, the comparison to the multitude of *Rhizopus* fermentations reported in literature is poor [5]. *Rhizopus oryzae* (ATCC 20344, also referred to as *Rhizopus delemar*) is one of the most prominent and studied *Rhizopus* species for producing fumarate. Genetic modifications on *R. oryzae* have resulted in small differences in the fermentation outcome [12, 13], but most open literature studies employ the wild strain where fumarate titres ranging from 25 to $103\,\hbox {g}\,\hbox {L}^{-1}$ and volume-based productivities ranging from 0.19 to $1.21\,\hbox {g}\,\hbox {L}^{-1}\,\hbox {h}^{-1}$ are commonly obtained. For a comprehensive review on fermentative fumarate production consult the paper by Sebastian [5].

*Rhizopus oryzae* excretes fumaric acid under nitrogen-limited conditions with the co-production of ethanol typically observed in this ’non-growth’ production phase. Ethanol is an unwanted by-product that reduces fumarate yield and should be minimised from a processing perspective. Numerous authors attribute ethanol formation to anaerobic zones within the fungal mycelium [14–16]. *R. oryzae* is a facultative anaerobe that can survive under anaerobic conditions [17] where the ethanol pathway is used for generating intracellular ATP. Most *R. oryzae* fermentations employ suspended biomass pellets and numerous efforts have been made to reduce the pellet diameter by manipulating pH, inoculum size, nitrogen source and glucose concentration [16, 18, 19]. The postulate that reduced pellet diameters will decrease anaerobic zones within the mycelium matrix and hence reduce ethanol formation has been stated but has never been conclusively proven. Accordingly an uncertainty exists with regards to the influence of oxygen availability on ethanol formation.

Organisms like the yeast *Saccharomyces cerevisiae* are known to produce ethanol under full aerobic conditions [20]. The phenomena, referred to as the Crabtree effect [21] is characterised by the formation of ethanol when ample glucose is available in the extracellular environment. Crabtree-positive organisms have the ability to consume glucose at a rate faster than the corresponding maximum respiratory rate, whereby excess carbon exits in the form of ethanol. The overflow mechanism is independent of the oxygen supply rate to the cell. Commercial production of baker’s yeast (*S. cerevisiae*) targets maximum biomass yield on glucose and attempts to avoid the formation of ethanol in an aerobic fermenter. This is achieved by controlling the glucose feed rate to the fermenter in order to manipulate the maximum cellular uptake rate of glucose. The fed-batch scheme is successful in avoiding ethanol formation as long as the uptake rate of glucose results in a respiratory carbon flux less than the maximum [22]. Glucose uptake rates are dependent on the extracellular glucose concentration and ethanol overflow can be avoided at concentrations below $150\,\hbox {mg}\,\hbox {L}^{-1}$ [23].

**Fig. 1:**
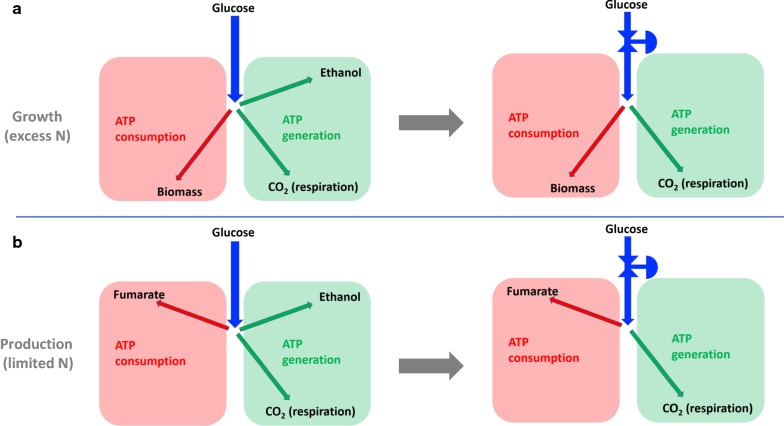
The postulated effect of glucose limitation on growth and fumarate production fermentations. Glucose uptake rates are controlled via fed-batch fermentation and are indicated with a reducing valve on the incoming glucose flux. It is postulated that glucose throttling will reduce ethanol formation in both growth and fumarate production fermentations

It is postulated in this study that *R. oryzae* is a Crabtree-positive organism and that ethanol formation can be avoided by manipulating cellular glucose uptake rates. *R. oryzae* fermentations typically consist of a growth and fumarate production stage [24] where the growth stage shares a similarity with the production of baker’s yeast. In Fig. 1a it is illustrated how glucose flux is balanced between energy consumption and energy production pathways. For growth conditions where excess nitrogen is supplied two fermentation strategies can be employed, one where the glucose uptake rate is regulated by the organism (batch fermentation) and the other where the glucose uptake rate is controlled by selective glucose addition (fed-batch fermentation). Figure 1 indicates selective addition of glucose via a reducing valve on the glucose flux. It is postulated that by throttling the glucose addition ethanol production should disappear since the respiratory capacity is sufficient to process all catabolic carbon. For production conditions under limited nitrogen supply a similar scenario exists where glucose uptake can be regulated via fed-batch fermentation. Here the energy consuming pathway cannot be the formation of biomass since nitrogen absence prohibits protein formation. Fumarate excretion from the cell is known to be energy intensive where the amount of ATP required by the acid transporter depends on the extracellular pH [25]. Under nitrogen limitation fumarate export thus replaces biomass formation as a energy consuming pathway and a likely scenario exists where ethanol overflow can be suppressed under these conditions.

In this study, an immobilised form of *R. oryzae* is used to investigate the organism’s Crabtree characteristics. We have successfully employed the immobilised process in previous studies [24, 26, 27] and have shown that biomass-medium separation and process up-scaling are considerably simplified when compared to systems with suspended fungal pellets. The initial experimental work explores the effect of oxygen availability in the medium on ethanol formation, while subsequent experiments investigate the effect of glucose limitation on the growth and fumarate production (nitrogen limited) response in order to test the postulated response depicted in Fig. 1b.

## Results and discussion

### Growing biomass in excess glucose

**Fig. 2:**
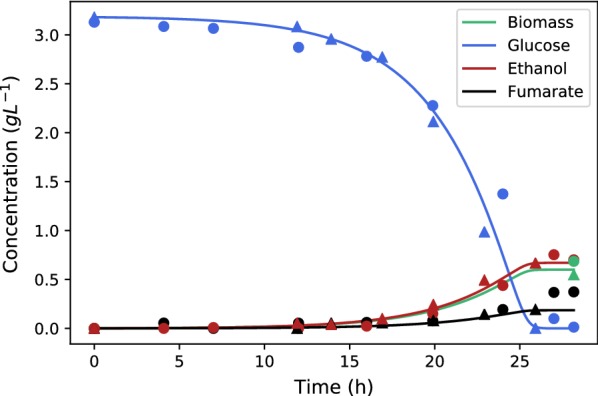
Repeat profiles of metabolite accumulation under growth conditions using 3.1 g/L of glucose and nitrogen excess. The circles and triangles identify the two repeat runs. Notable production of ethanol and fumarate was observed, with $0.62\,\pm \,0.097\hbox {g}\,\hbox {L}^{-1}$ of biomass obtained at the end of the run. Fitted model indicate biomass accumulation up to the final measured point. The model employed fixed yield coefficients of ethanol and fumarate on glucose (0.211 and 0.058, respectively). The estimated maximum specific growth rate was found to be $0.255\,\hbox {h}^{-1}$ and the Monod constant was $0.176\,\hbox {g}\,\hbox {L}^{-1}$

All fermentations consisted of a growth and a production phase. Growth was always performed with excess nitrogen in order to establish the biomass that will be used for fumarate production. Production always occurs after growth in a limited nitrogen environment to trigger fumarate excretion. Figure 2 depicts the metabolite concentration profiles obtained under growth conditions for 2 repeat fermentations at a pH of 5. Complete consumption of all glucose (3.1 g) occurred within $25.6\pm 1.8\hbox {h}$. Complete consumption was longer than the reported period if NaOH was used for initial pH correction prior to inoculation. The biomass yield on glucose was found to be $0.196\pm 0.033\hbox {g}\,\hbox {g}^{-1}$.

The fermentation profile in Fig. 2 starts with an extended lag phase after which glucose consumption occurs rapidly. Notable amounts of ethanol and fumarate formed during the fermentation with the final mass yield of ethanol higher than that of biomass. Malic acid, succinic acid and pyruvic acid were also produced, but only in trace concentrations. A model was fitted to estimate the growth rate of *R. oryzae*. Constant yield coefficients on glucose were used for ethanol and fumarate, there values were 0.211 and 0.058, respectively, this resulted in a reasonable fit as observed in Fig. 2. The specific growth rate was estimated to be $0.255\,\hbox {h}^{-1}$.

The extensive ethanol produced, despite fully aerobic conditions, reminds one of aerobic growth of *S. cerevisiae*. The results in Fig. 2 therefore suggest that *R. oryzae* might be a Crabtree-positive organism where the respiratory capacity reaches a maximum under excess glucose supply. The ethanol formed during the fermentation can accordingly be interpreted as an overflow from glycolysis, where additional ATP is obtained via ethanol fermentation. Interestingly a small amount of fumarate also forms during the fermentation despite excess nitrogen conditions. From an energetic point of view the ethanol and fumarate excretion will counter each other since 3 ATPs are required to excrete a mole of fumarate at a pH of 5 [25], while ethanol will produce only 1 mole of ATP per mole of ethanol. Given the final mole ratio of ethanol to fumarate of 3.05, it appears that most ATP generated via ethanol production is used for fumarate excretion, indicating that there is little energy gained from the ethanol overflow when compared to the aerobic batch growth of *S. cerevisiae*.

### Fumaric acid production with DO variation

The results in Fig. 2 suggest that *R. oryzae* might be a Crabtree-positive organism. Other authors [14, 15] suggest an alternative explanation for ethanol formation, where anaerobic zones within fungal pellets are the reason for ethanol production. In order to examine the alternative explanation an experiment was performed where the DO in the fermenter was significantly increased from 18.4 to 85% under production conditions where significant fumarate excretion occurs. The premise of the experiment is that the higher partial pressure of oxygen will eradicate anaerobic zones, since the driving force for oxygen mass transfer will be increased by a factor of 4.6. The external liquid oxygen tension (DO) was found to be proportional to the partial pressure indicating fast gas to liquid mass transfer.

**Fig. 3:**
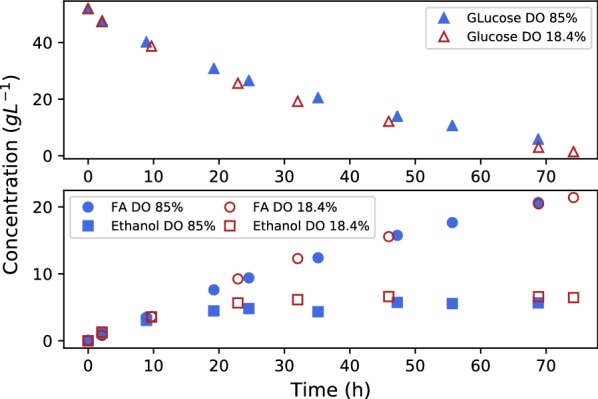
The effect of DO on fumarate production where $50\,\hbox {g}\,\hbox {L}^{-1}$ of glucose was initially used. The DO was varied from 18.4 to 85%. A negligible difference was observed between the two runs

The results of the two runs at different DO values can be seen in Fig. 3. Note that growth similar to the results in Fig. 2 preceded the reported production phase. The biomass produced during the growth phase has a thickness of 1 mL to 2 mL, similar to ideal pellet diameters as reported in literature [14, 18]. From Fig. 3 it is clear that the production characteristics of *R. oryzae* is unaffected by the dissolved oxygen concentration, with ethanol, fumarate and glucose profiles exhibiting almost identical behaviour.

The notion that biomass morphology relates to the extent of ethanol formation in *R. oryzae* is often used [14, 15]. However, the production of ethanol has not been negated as a result of varied morphology in any study. The results from the experiment in Fig. 3 provide evidence that perceived anaerobic zones do not exist in the mycelium matrix when the depth of the matrix is 2 mm or less. It is clear that improved oxygenation within the matrix has zero effect on the ethanol response. This observation provides further evidence of the Crabtree-positive characteristics of *R. oryzae*, where ethanol production or overflow is unrelated to oxygen availability.

### Manipulating glucose uptake rates under growth conditions

The results from Figs. 2 and 3 provide preliminary evidence of the Crabtree-positive nature of *R. oryzae*. In order to comprehensively verify the observation, a link should be made between the glucose uptake rate and the ethanol excretion rate. Aerobic ethanol formation with *S. cerevisiae* is typically negated by reducing the glucose uptake rate [28]. This is achieved by maintaining low concentrations of glucose in the extracellular space. The Monod effect describes a low concentration regime where substrate uptake rates are proportional to substrate concentration. At higher substrate concentrations, a zero-order regime is typically observed where substrate concentration has no influence on uptake rate. Accordingly, glucose uptake rates can be manipulated by operating the fermenter at low glucose concentrations. This can be achieved by a fed-batch fermenter where the glucose addition rate is operated at a similar value to that of the volumetric glucose consumption rate [22]. In the following sections the fed-batch strategy will be employed on both growth and production of *R. oryzae* in order to see if the perceived Crabtree characteristics can be utilised to minimise ethanol production.

**Fig. 4:**
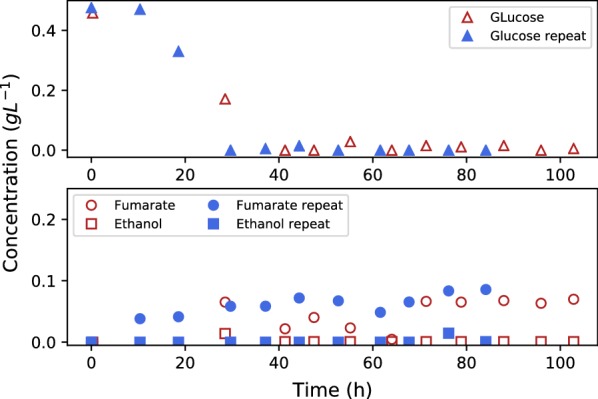
Glucose, ethanol and fumarate concentrations during fed-batch growth of *R. oryzae*. Glucose was added at a constant rate of $0.07\,\hbox {g}\,\hbox {L}^{-1}\,\hbox {h}^{-1}$. All concentrations approximate zero, except glucose in the initial stages of the experiment. Ethanol overflow was clearly avoided

Figure 4 presents the glucose, ethanol and fumarate profiles for the growth run where glucose was added at a constant rate of $0.07\,\hbox {g}\,\hbox {L}^{-1}\,\hbox {h}^{-1}$. Zero glucose was present in the growth medium prior to spore inoculation. It is clear that ethanol formation is completely avoided by the slow supply of glucose. Trace amounts of fumarate were present from the start of the fermentation with negligible additional amounts formed during the fermentation. The glucose concentration also remained close to zero, except right at the beginning where initial growth was unable to consume all the supplied glucose. The situation rectified at 30 h once biomass accumulation increased in the fermenter. The slight overshoot in the initial glucose concentration ($0.46\,\hbox {g}\,\hbox {L}^{-1}$) was not accompanied by ethanol overflow, hinting that the glucose uptake rate was always less than the corresponding maximum respiratory rate. The results are in close agreement with glucose controlled *S. cerevisiae* growth, suggesting that the Crabtree traits of R .oryzae can be utilised to reduce ethanol formation.

### Manipulating glucose supply under production conditions

The results presented thus far provides clear evidence of the Crabtree characteristics of *R. oryzae* under growth conditions. The main objective of the fermentation is to produce fumarate and not biomass. Accordingly, the Crabtree response should be tested under production conditions where the nitrogen supply is limited. For this phase of the fermentation the energy consumption within the cell will be predominantly used for exporting fumarate where 3 moles of ATP are required to export one mole of fumarate [25]. The export cost under production conditions will effectively replace the energy expenditure used for biomass synthesis in the growth phase of the fermentation. The main question is whether the overflow mechanism under production conditions emulate that of the growth conditions and whether the main carbon metabolism reacts in a similar manner. To address this question, the same glucose feed rate employed under glucose limited growth conditions were used ($0.07\,\hbox {g}\,\hbox {L}^{-1} \,\hbox {h}^{-1}$) from the start of the production phase. Zero fumarate (or ethanol) production was observed for 80 h, indicating that all glucose was respired to obtain energy for the cellular transition from growth metabolism to production metabolism. The transition period for production fermentations with excess glucose ($50\,\hbox {g}\,\hbox {L}^{-1}$) typically takes 20 h after which fumarate production commences [24]. Accordingly, it was decided to increase the initial glucose addition rate to $0.131\,\hbox {g}\,\hbox {L}^{-1}\,\hbox {h}^{-1}$ to see whether the transition period could be reduced. In this experiment fumarate production commenced after 20 h and consequently this glucose addition rate was employed as the minimum value in further experiments.

**Fig. 5:**
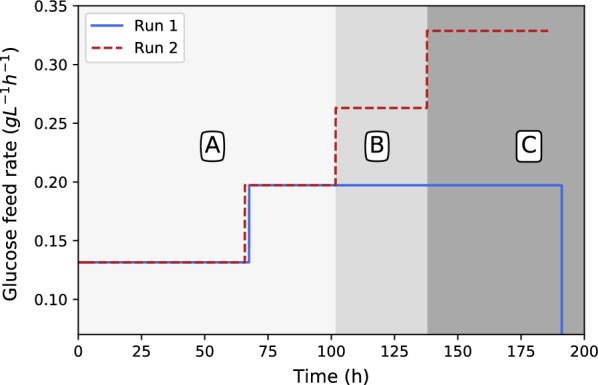
Glucose dosing rates for run 1 and 2. The dosing rate of run 1 was increased by 50% once. Towards the end of the fermentation, dosing was stopped and the glucose concentration was depleted. The dosing rate of run 2 was increased three times by 50% of the original rate

Two extended production fermentations were performed for approximately 200 h each. Both fermentations employed a growth strategy similar to the results presented in Fig. 2. The only difference between the two runs is the glucose addition strategies as can be seen in Fig. 5. Both runs initiated with the minimum glucose feed rate of $0.131\,\hbox {g}\,\hbox {L}^{-1}\,\hbox {h}^{-1}$ up to 66 h when the feed rate was increased by 50% ($0.197\,\hbox {g}\,\hbox {L}^{-1} \,\hbox {h}^{-1}$).

**Fig. 6:**
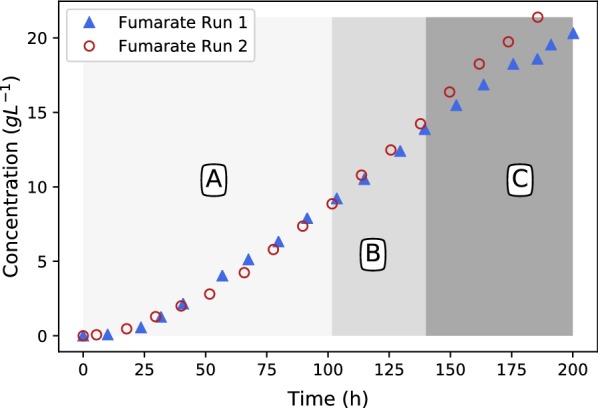
Fumarate production profiles for runs 1 and 2. Note the slight increase in fumarate excretion rates in regimes B and C

**Fig. 7:**
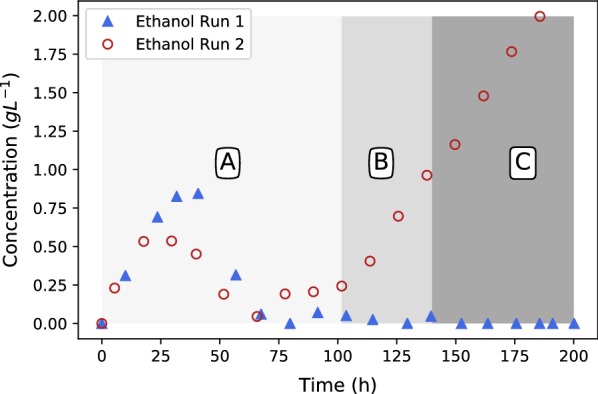
Ethanol profiles for runs 1 and 2. Beyond the first 25-h transition phase, no ethanol overflow is observed in run 1. Run 2 exhibits clear ethanol overflow in regimes B and C where glucose addition rates were increased

**Fig. 8:**
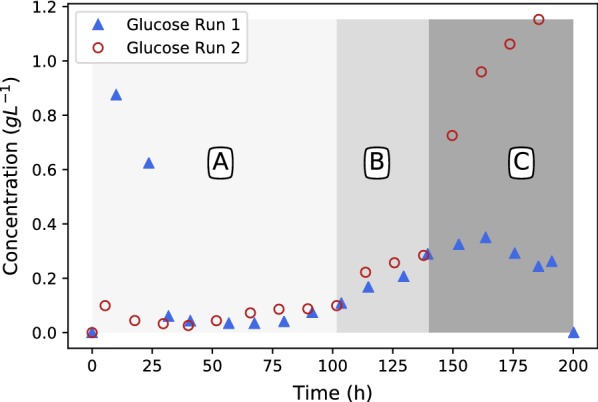
Glucose profiles for runs 1 and 2. Glucose breakthrough is observed for run 2 in regime C, where glucose addition rate exceeds the glucose consumption rate

Run 1 remained at this value for the remainder of the fermentation while run 2 had two subsequent increases (see Fig. 5). The fumarate, ethanol and glucose response of the two runs can be seen in Figs. 6, 7 and 8.

The experimental conditions of run 1 and 2 for the first 100 h were identical and hence presents a repeat run. Figures 6, 7 and 8 clearly show good repeatability with regards to the metabolic response. The ethanol response of run 1 indicates a regime after 75 h where zero ethanol is produced. These results are in alignment with the results presented in Fig. 4 (fed-batch growth) and indicates that the metabolic overflow to ethanol can be completely avoided at a glucose addition rate of $0.131\,\hbox {g} \,\hbox {L}^{-1}\,\hbox {h}^{-1}$. It is clear from these results that ethanol excretion can be avoided by controlling the glucose addition rate. During the first 75 h of production an ethanol peak was observed, indicating initial production followed by the metabolic consumption of ethanol. This implies that the transition period (first 25 h) is associated with a slight oversupply of glucose and hence the initial ethanol production. The behaviour switches once fumarate production commences and ethanol consumption is observed between 25 and 75 h. This indicates that once the production mode is established at around 25 h, the supplied glucose rate on a biomass basis is less than the corresponding maximum respiratory rate and accordingly ethanol breakdown can be accommodated within the mitochondria. This is further illustrated by the first step-up in glucose addition at 75 h, where zero ethanol formation is observed despite an increase in cellular glycolytic flux. Both results suggest that a feed rate of $0.131\,\hbox {g}\,\hbox {L}^{-1}\,\hbox {h}^{-1}$ corresponds to a glycolytic flux below the ethanol overflow point.

When looking at the complete duration of run 1 a constant fumarate production rate of $0.15\,\hbox {g}\,\hbox {L}^{-1}\,\hbox {h}^{-1}$ is observed after 75 h (see Fig. 6). Given the glucose supply rate $0.197\,\hbox {g}\,\hbox {L}^{-1} \,\hbox {h}^{-1}$ it is evident that this rate also corresponds to a glycolytic flux below the ethanol overflow point. When considering the glucose concentration profile in Fig. 8, a slight glucose increase is observed towards the end, with a maximum value of $0.28\,\hbox {g}\,\hbox {L}^{-1}$ obtained at 175 h. It is evident that this glucose concentration is below the overflow threshold concentration and is in close agreement to the value of $0.15\,\hbox {g}\,\hbox {L}^{-1}$ reported for *S. cerevisiae* [23]. Run 1 clearly illustrate that the Crabtree effect of *R. oryzae* can be utilised to avoid ethanol by-product formation, a very useful result for further development of the *R. oryzae* fumaric acid process.

Run 2 was performed to establish the ethanol overflow and glucose breakthrough point. The results of the first 100 h of run 2 were in good agreement with that of run 1, indicating repeatability in the method. The first deviation between run 1 and run 2 occurs at 100 h when the glucose addition rate was increased to $0.263\,\hbox {g}\,\hbox {L}^{-1}\,\hbox {h}^{-1}$ in run 2 while the glucose addition rate of run 1 remained at a feed rate of $0.197\,\hbox {g}\,\hbox {L}^{-1}\,\hbox {h}^{-1}$ for the remainder of the run. Figures 6, 7 and 8 are mapped with 3 separate regimes A, B and C. Regime A represents similar operation between run 1 and run 2, regime B represents the time when run 2 glucose feed rate is 33% higher than in run 1 and regime C represents the time when run 2 glucose feed rate is 67% higher than in run 1.

The major difference between run 1 and 2 in regime B can be seen in the ethanol profiles of Fig. 7. It is clear that ethanol overflow starts occurring when the glucose feed rate is increased to $0.263\,\hbox {g}\,\hbox {L}^{-1}\,\hbox {h}^{-1}$. The glucose uptake rate in regime B exceeds that of the carbon flux that can be accommodated by respiration and fumarate excretion, resulting in the excess carbon overflowing as ethanol. From Fig. 8 it can be observed that the glucose profiles of run 1 and run 2 remain similar, hinting that almost all of the glucose fed is consumed when the glucose concentration is below the threshold value of $0.28\,\hbox {g}\,\hbox {L}^{-1}$. In Fig. 6 it can be seen that the fumaric acid profile of run 2 starts exhibiting a steeper upward gradient once regime B commences. The difference, however, is clearly observed in regime C. This observation suggests that the ethanol overflow point (indicated by the glucose addition rate) is slightly higher than $0.197\,\hbox {g}\,\hbox {L}^{-1}\,\hbox {h}^{-1}$ although less than $0.263\,\hbox {g}\,\hbox {L}^{-1}\,\hbox {h}^{-1},$ where ethanol overflow clearly occurs. The increase in fumarate excretion in regime B can be calculated using the fumarate concentration measurements or the NaOH dosing rates in regime B where the trace amounts of succinic and malic acid formed are subtracted from the neutralisation calculation. Given these two calculations the ethanol overflow point was calculated to occur at a glucose feed rate of $0.244\,\hbox {g}\,\hbox {L}^{-1}\,\hbox {h}^{-1}$.

In regime C the glucose feed rate was increased to $0.329\,\hbox {g}\,\hbox {L}^{-1}\,\hbox {h}^{-1}$. From Fig. 7 it is clear that the ethanol overflow further increases, indicating that additional glucose uptake by *R. oryzae* is diverted to ethanol. Figure 8 shows a major deviation between the glucose profiles of run 1 and 2. It is evident that glucose breakthrough occurs in run 2, implying that the addition rate of glucose exceeds the uptake rate of *R. oryzae*. The maximum glucose uptake rate accordingly lies somewhere between 0.263 to $0.329\,\hbox {g}\,\hbox {L}^{-1}\,\hbox {h}^{-1}$ and additional experimental work is required to quantify the maximum uptake rate more accurately.

When defining and quantifying the maximum glucose uptake rate (breakthrough rate) as well as the glucose uptake rate where ethanol excretion commences (overflow rate), it is preferable to use a biomass basis as opposed to the volumetric basis used in the discussion above. Quantification of biomass can only be performed at the end of the production run where $2.49\,\hbox {g}\,\hbox {L}^{-1}$ was obtained for run 1 and $2.51\,\hbox {g}\,\hbox {L}^{-1}$ obtained for run 2. These amounts differ significantly from the biomass amounts obtained using the growth procedure in Fig. 2 where an average of $0.617\,\hbox {g}\,\hbox {L}^{-1}$ of biomass was obtained. It should be noted that this growth procedure is the exact same as that run 1 and 2 utilised. The major increase in biomass during the production phase is unexpected given that the total urea fed over the production period was only $0.116\,\hbox {g}\,\hbox {L}^{-1}$. Based on the general biomass formula $\hbox {CH}_{1.8}\hbox {O}_{0.5}\hbox {N}_{0.2}$ [29], additional protein synthesis as a result of urea will only contribute 4% of additional biomass and cannot explain the fivefold increase in biomass. It is accordingly suspected that carbohydrate accumulation in the biomass is the major reason for the mass increase. Given this it was decided to quantify the biomass-based overflow and breakthrough rates using the biomass amount obtained after growth, where the protein contents of the cell are much higher than compared to the spent production biomass. Accordingly, the ethanol breakthrough rate is calculated to be $0.395\,\hbox {g}\,\hbox {g}^{-1}\,\hbox {h}^{-1}$ while the glucose break through rate lies between 0.426 and $0.533\,\hbox {g}\,\hbox {g}^{-1}\,\hbox {h}^{-1}$.

Towards the end of run 1 a yield of $0.802\,\hbox {g}\,\hbox {g}^{-1}$ fumarate on glucose was obtained, this was over 50 h. The yield over the entire fermentation was $0.713\,\hbox {g}\,\hbox {g}^{-1}$ since fumarate was not produced during the first hours of the fermentation. The yield for run 2 during the highest glucose feed rate was found to be $0.596\,\hbox {g}\,\hbox {g}^{-1}$. This translates to a $0.206\,\hbox {g}\,\hbox {g}^{-1}$ improvement in the yield for run 1 as a result of controlling the glucose addition below the ethanol breakthrough rate. This illustrates the effectiveness of the fed-batch reactor operation and the extent of carbon losses to ethanol that occur under batch conditions. These yields were calculated by accounting for all fumarate produced over the period and the amount of glucose added to the reactor.

## Conclusion

The production of fumarate using *Rhizopus oryzae* has always been associated with the simultaneous production of ethanol. It was hypothesised that the production of ethanol was as a result of the Crabtree effect, a common overflow mechanism in yeasts. The production of ethanol was previously attributed to anaerobic zones within the mycelium matrix. Experiments with a fivefold increase in liquid oxygen tension resulted in similar ethanol responses suggesting that ethanol formation occurs under fully aerobic conditions within the fungal matrix.

Ethanol production by *S. cerevisiae* has been successfully negated using closely controlled glucose addition [22]. This method was employed in a biomass growth experiment. It was found that a glucose feed rate of $0.07\,\hbox {g}\,\hbox {L}^{-1}\,\hbox {h}^{-1}$ resulted in no ethanol production. These results indicated that *R. oryzae* is a Crabtree-positive organism since ethanol production was negated by manipulating the glucose feed rate.

The same fed-batch strategy was then tested for the nitrogen-limited fumarate production phase. A glucose feed rate was found that produced no ethanol while still producing fumarate. The ethanol overflow point was estimated to occur at a glucose feed rate of $0.395\,\hbox {g}\,\hbox {g}^{-1}\,\hbox {h}^{-1}$, while a range for the maximum glucose uptake rate was found.

The findings illustrate that fumarate can be produced without the co-production of ethanol. The term “homofumarate production” is used to describe the condition where all carbon exits the cell as either fumarate or respiratory $\hbox {CO}_{2}$. The result provides new insights toward developing a high-yield industrial process to produce fumaric acid with *Rhizopus oryzae*.

## Methods

### Microorganism and culture conditions

*Rhizopus oryzae* (ATCC 20344) was used for all fermentations and was prepared as described by Naude and Nicol [27]. The spore inoculum used for batch growth fermentations had a spore concentration of $8\times 10^{6}\,\hbox {mL}^{-1}$ of which 10 mL were aseptically injected into the reactor through a silicon septum.

### Medium

Varying amounts of glucose and urea were added to a mineral medium for all fermentations. The mineral medium contained (all of the following values have units of $\hbox {g}\,\hbox {L}^{-1}$): $0.6 \,\hbox {KH}_{2}\hbox {PO}_{4}$, $0.25\,\hbox {MgSO}_{4}\cdot 7\hbox {H}_{2}\hbox {O}$, $0.088\,\hbox {ZnSO}_{4}\cdot 7\hbox {H}_{2}\hbox {O}$ and $0.005\,\hbox {FeSO}_{4}\cdot 7\hbox {H}_{2}\hbox {O}$. Biomass was grown under batch conditions with $3.1\,\hbox {g}\,\hbox {L}^{-1}$ glucose and $2.0\,\hbox {g}\,\hbox {L}^{-1}$ urea [27]. The medium for fed-batch growth of biomass contained the $2.0\,\hbox {g}\,\hbox {L}^{-1}$ urea but no glucose at the beginning of the fermentation as this was fed continuously at a rate of $0.07\,\hbox {g}\,\hbox {L}^{-1}\,\hbox {h}^{-1}$. The batch production fermentations contained $50\,\hbox {g}\,\hbox {L}^{-1}$ glucose and $0.1\,\hbox {g}\,\hbox {L}^{-1}$ urea. The fed-batch production fermentations began with only the mineral solution, urea was fed at a rate of $0.625\,\hbox {mg}\,\hbox {L}^{-1}\,\hbox {h}^{-1}$ and glucose fed at a rate between 0.131 and $0.329\,\hbox {g}\,\hbox {L}^{-1}\,\hbox {h}^{-1}$. In order to achieve low dilution rates high concentration solutions of both glucose and urea were made with $342\,\hbox {g}\,\hbox {L}^{-1}$ and $16\,\hbox {g}\,\hbox {L}^{-1}$, respectively. The dilution rate for the fed-batch production fermentations varied between 0.0018 and $0.0027\,\hbox {h}^{-1}$, taking into account the glucose and urea additions, as well as the NaOH dosing. The urea solution incorporated the mineral solution to ensure that the mineral composition in the reactor remained constant over the duration of the experimental run. All the solutions were sterilised at $121\,^{\circ }\text {C}$ for 60 min. All chemicals used were obtained from Merck (Modderfontein, South Africa).

### Reactor operation

The reactor design was adapted from a previous study which researched fumaric acid production with *R. oryzae* [27]. The reactor has a liquid volume of 1.08 L and a gas volume of 0.380 L. This design incorporates a textured polypropylene tube in the centre of the glass reactor tube, serving as an attachment surface for *R. oryzae* during the growth phase. Once the biomass has been grown, the immobilised fungus can be rinsed with a mineral medium containing no nitrogen. The biomass produced has a thickness of approximately 1 mm to 2 mm and covers an area of $97.14\,\hbox {cm}^{2}$. The benefits of this system for biomass production over a mobilised pellet approach is that the thickness of the biomass can be closely controlled by means of the initial glucose concentration in the growth medium. Immobilised biomass also allows a simple sterile transition from growth to production conditions since the medium can be easily drained and replaced at the end of the growth phase. The switch to production requires rinsing of the biomass to remove residual nitrogen from the reactor. This was done by washing twice with the nitrogen free mineral solution. Once this has been completed the reactor is filled with the production phase medium and adjusted to a pH of 5. Growth and production fermentations were controlled at a pH of 5 using a $10\,\hbox {mol}\,\hbox {L}^{-1}\hbox {NaOH}$ solution as a neutralising agent.

The concentrated glucose and urea solutions were fed through 0.5 mm marprene tubing with the 120U Watson-Marlow (Johannesburg, South Africa) peristaltic pump. This allowed for fine control of the feed flow rate between $0.11\,\hbox {mL}\,\hbox {h}^{-1}$ to $225\,\hbox {mL}\,\hbox {h}^{-1}$ with increments of $0.11\,\hbox {mL}\,\hbox {h}^{-1}$. The reactor was fed a gas mixture that consisted of 8% $\hbox {CO}_{2}$, with 18.4% $\hbox {O}_{2}$ and $\hbox {N}_{2}$ making up the complement, in all fermentations, unless otherwise stated. This mixture was controlled using an SLA5850 mass flow controller from Brooks (Hatfield, USA). The gas and liquid phases of the reactor were recycled to ensure no concentration gradients were present, as described by Naude et al. [27].

### Analytical methods

The fermentations were sampled at regular intervals over the period of the fermentation, with shorter experiments being sampled more frequently for a higher resolution. HPLC was used to determine the concentrations of glucose, fumarate, ethanol, malic acid and succinic acid in the samples as described by Naude et al. [27]. The dry cell mass was determined at the end of each experimental run. The immobilised biomass was removed from the polypropylene tube and centrifuged at 2000 rpm for 10 min, the supernatant was re-suspended in distilled water and the biomass was then centrifuged again. This was repeated a total of three times. The biomass was finally dried at $70\,^{\circ }\hbox {C}$ for 48 h before being weighed.

## Data Availability

The datasets analysed during the current study are available from the corresponding author on reasonable request.

## Abbreviations

ATCC: American Type Culture Collection
ATP: Adenosine triphosphate
DO: Dissolved oxygen
HPLC: High-performance liquid chromatography
TCA: Tricarboxylic acid cycle
